# Evaluating psychosocial and physical activity outcomes following an intervention among Filipino Americans

**DOI:** 10.34172/hpp.2021.26

**Published:** 2021-05-19

**Authors:** Aisha Bhimla, Ksenia Power, Michael Sachs, Allegra Bermudez, Jessica Dinh, Nicholas San Juan, Grace X. Ma

**Affiliations:** ^1^Department of Kinesiology, College of Public Health, Temple University, Philadelphia, PA, USA; ^2^Center for Asian Health, Lewis Katz School of Medicine, Temple University, Philadelphia, PA, USA; ^3^College of Public Health, Temple University, Philadelphia, PA, USA; ^4^Rowan University, Glassboro, NJ, USA; ^5^Department of Clinical Sciences, Lewis Katz School of Medicine, Temple University, Philadelphia, PA, USA

**Keywords:** Asian American, Physical activity, Community-based

## Abstract

**Background:** Physical activity (PA) is a strong contributor to enhancing a healthy lifestyle and preventing numerous chronic diseases. As ethnic minorities engage in low levels of PA, psychosocial and activity-based interventions for sustaining PA are crucial.

**Methods:** The 6-month intervention incorporated culturally tailored educational workshops and weekly PA classes at a community center. Educational workshops were led by six trained community health workers (CHWs). Participants (n=37) completed pre- and post-intervention questionnaires regarding PA related self-efficacy, outcome expectations, social support, enjoyment, self-regulation, goal setting, and overall PA.

**Results:** Following the intervention, study participants exhibited increases in weekly PA levels. Wilcoxon Signed-Rank test revealed higher median scores for Exercise Self-Efficacy Scale (ESES), Identified Regulation, and Intrinsic Motivation. Positive changes were observed for Physical Outcome Expectations, Social Outcome Expectations, Self-Evaluative Outcome Expectations, Physical Activity Enjoyment, Social Support for Exercise Scale – Family, Social Support for Exercise – Friends, and Exercise Goal-Setting.

**Conclusion:** Community-based PA interventions may provide potential benefits to Filipino Americans, an ethnic Asian minority group, in identifying exercise benefits, developing proper exercise goals, increasing motivation, promoting PA behavior, and facilitating long-term PA adherence.

## Introduction

Physical activity (PA) is an essential element to promoting a healthy lifestyle and mitigating the development of numerous chronic diseases, including cancer, heart disease, hypertension, diabetes, and obesity.^1-5^ Over the past few decades, the United States (US) population has continued to face degrading levels of PA, with only one in four persons meeting the Centers for Disease Control and Prevention (CDC) guidelines of 150 minutes of moderate intensity activity and two days of muscle strengthening activities per week.^6,7^ Strategies to promote and sustain PA behaviors have been widely implemented, from individualized programs and best practices among patients and health individuals to improving the environments, communities, and policies which promote participation in PA.^4,5,8^

It has been widely established in the literature that immigrant and ethnic minority communities face lower levels of participation in PA.^9-11^ Among them, Filipino Americans represent one of the largest Asian American immigrant populations in the United States that report low rates of PA.^12-14^ Ethnic minority and immigrant groups are known to underreport their levels of PA specifically on self-report measures.^15^ For instance, despite the similarity in the rates of PA on objective measures, South Asian populations reported around 20 minutes less of PA per day than the White counterparts.^15^ Thus, the way in which PA is defined and viewed by ethnic minority groups can be influenced by certain socio-cultural factors beyond total frequency and intensity of PA. ^15^

Population and community-based studies have reported that 25%-33.3% of Filipinos met the recommended PA guidelines of 150 minutes of moderate or 75 minutes of vigorous PA per week.^12,16-19^ This is disparagingly low in comparison to the national average of 49% of Americans meeting the CDC guidelines for aerobic PA in 2015.^20^ Recently, a published study on PA among Asian subgroups in California found that middle-aged Filipinos had the lowest odds of meeting the American College of Sports Medicine (ACSM) PA recommendations compared to five other ethnic groups and non-Hispanic Whites.^19^

Despite representing one of the largest immigrant populations in the US, the health conditions and behaviors of this population are poorly understood. Filipinos have faced significantly higher rates of heart disease, hypertension and type 2 diabetes, compared to non-Hispanic whites and other Asian groups.^14,16,17,21-24^ Promoting PA in this population is strongly needed towards combating chronic disease development in Filipinos. Filipino Americans indicate that they face numerous psychosocial barriers to being physically active, including a lack of knowledge regarding PA benefits, lack of support from family and friends, low motivation, and low self-efficacy.^24-27^ Thus, psychosocial factors may impede Filipinos’ participation in PA and strategies are needed to increase PA, especially among this ethnic minority population.

Psychosocial and activity-based interventions, aimed to increase participants’ motivation, have shown potential for efficacy in promoting PA among minority groups.^28-30^ These interventions include self-report measures of psychosocial factors influencing one’s PA engagement (e.g., PA motivation, PA enjoyment, perceived benefits, and PA outcome expectations), as well as an activity-based component (e.g., educational workshops, supervised training sessions). Theoretical constructs that drive these interventions have included the Social Cognitive Theory (SCT), which accounts for individual, environment, and behavior and places self-efficacy as the main construct.^31^ Previous findings indicate that healthy minority adults report significant improvements in fitness levels and PA adherence following supervised exercise sessions and educational workshops.^28-30^ These improvements are observed after short-term PA sessions typically occurring 2-3 times a week for 11-12 weeks. Better PA outcomes can also follow exercise variations, such as increased session frequency and intensity. Moreover, psychosocial and activity-based interventions in minority groups can be effectively utilized for weight gain prevention, which is linked with decreased risk of cardiovascular conditions.^28-30^ As ethnic minorities are more likely to engage in low levels of PA,^28-30^ psychosocial and PA-based interventions are necessary for successful behavior adoption and maintenance.

PA interventions among culturally and linguistically diverse (CALD) populations have constituted a wide range of methodological and program consideration.^29,30,32^ Several strategies have culturally adapted these interventions, which included using bicultural/bilingual research staff and personnel, hiring community health workers (CHWs), and adopting material and content that were linguistically and culturally tailored.^29^ In a systematic review of interventions focusing on CALD populations, positive changes in PA levels were observed following the intervention.^29^ Furthermore, CHWs have played a significant role in promoting PA among chronic disease at-risk populations, patients with chronic conditions, and healthy population.^33-36^ Thus, the use of CHWs is a successful intervention strategy to promote PA behavior among ethnic minority population.^29^

There is a significant gap in knowledge regarding how cultrally tailored interventions can improve PA levels among Asian Americans and need to understand how CHWs may play a role in PA adoption. The purpose of this study was to design, implement, and test Get Phit, a culturally tailored PA intervention, sensitive to the needs and preferences of Filipino Americans. Recruitment strategies, PA attitudes, beliefs, barriers, facilitators, and educational materials were identified through a rigorous formative research phase. Following the intervention, changes in PA levels and psychosocial outcomes related to PA were assessed. In this intervention, trained CHWs delivered the educational materials and led PA programs in a community-based setting.

## Materials and Methods

### Study setting and design

The entire study period, including planning (5 months), recruitment (3 months), program implementation (6 months), and data collection, occurred from July 2017 to August 2018 in the Greater Philadelphia Area of the United States. The study utilized a single group pre-test post-test design to determine the differences in PA levels and psychosocial outcomes related to exercise.

### Study procedures

The study was carried out by a collaboration between Temple University’s Department of Kinesiology and a Filipino Community Based Organization (CBO) located in Southern New Jersey. The CBO has members spanning across the Greater Philadelphia Area, including southern New Jersey. A research team of four members was created to develop the content and materials for the training program. The CHW training took place at the university, and data collection and workshops were held at the community center.

### Participants and recruitment

Participants were recruited from a population of approximately 500 community members attending the CBO in the southern New Jersey area. Recruitment of community members occurred from January to March of 2018. Implementation of the intervention lasted for 6 months, from March to August of the same year. To prevent potential participant loss due to seasonal weather changes, all educational workshops and PA classes were held indoors at the local community center. Also, the facility was located in close proximity to participants’ residence. Participants were recruited through announcements on social media, as well as flyers placed in the community center, sent through email, and posted in surrounding areas such as Filipino restaurants and venues.

No exclusion criteria were set for recruitment of community participants to engage in the outreach workshops and PA classes. However, prior to participating in the PA classes, participants completed the Physical Activity Readiness Questionnaire for Everyone (PAR-Q).^36^ This instrument is a short self-report questionnaire, which uncovers health and lifestyle issues of individuals prior to their engagement in an exercise program.^36^ If a participant answered yes to one or more questions, they were instructed to speak with their doctor about gaining permission to participate in the intervention, and whether the activities offered at the center as part of the study were safe and suitable for them to participate. Only two participants reported a medical condition, which precluded them from PA participation. Their responses were excluded from data analysis. Participants’ answers were confidential and only accessible to the research team. Participation was voluntary and could be discontinued any time.

### Conceptual framework

**Figure 1:**
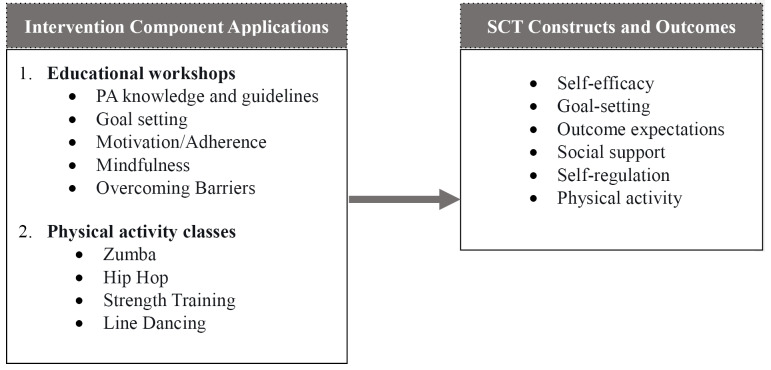
Get Phit intervention components, social cognitive theory constructs, and related PA outcomes.

This intervention incorporated aspects of health education, psychological skills implementation, and increased PA engagement in a community setting driven by the SCT.(Figure 1)^37^ This theory provides a deeper understanding of PA health behaviors, accounting for the interactions between individual, environment, and behavior.^38^ Self-efficacy, which is one of the main constructs of the SCT, is operationalized as the level of an individual’s confidence in his or her ability to independently execute a behavior. ^38^ High self-efficacy has been associated with successful adoption of healthy behaviors.^38^ The other constructs of the theory are task, planning, and coping self-efficacy, goal setting, and outcome expectations.^37-40^ Task self-efficacy is an individual’s confidence in his or her ability to independently perform certain parts of a task. Coping self-efficacy is an individuals’ confidence in his or her ability to perform a task under challenging conditions. Outcome expectations are a person’s social, physical, and self-evaluative outcomes, which serve as incentives (or disincentives) for PA engagement and adherence.^37^ Positive outcome expectations may include an individual’s belief in the benefits of PA, increased satisfaction and PA enjoyment, improved fitness levels, etc.^37^ Positive outcome expectations have shown to be an important factor for engaging in health behaviors.^37^ Finally, self-regulation includes individual behaviors necessary for monitoring and reinforcing PA engagement (e.g., goal-setting, PA planning and tracking).^37^ The modeling of the SCT constructs highly influences PA engagement, planning, and adherence.^37^

A meta-analysis on the utilization of the SCT in determining PA behavior showed that increased self-efficacy is positively associated with PA behavior.^37^ Ultimately, each psychosocial construct within the SCT interacts in a mediating fashion; higher self-efficacy results in positive outcome expectations regarding PA benefits, as well as leads to higher goal-setting and a greater ability to overcome PA barriers, thus resulting in greater PA levels. The SCT constructs were incorporated in the intervention delivery and materials and their impact was assessed pre- and post-intervention.

### Get Phit: a community led physical activity intervention

A total of six CHWs were recruited for the intervention delivery. Inclusion criteria for the CHWs included: being over 18 years old, self-identifying as Filipino, having a general health professional/allied health background, and being regularly physically active. The CHWs then underwent four sessions of training held at Temple University. The training involved learning the educational materials to carry out the intervention with community members.

The six-month Get Phit intervention delivered to the participants consisted of in-person educational workshops and various activities to promote PA. For the six-month period, from March to August of 2018, CHWs led monthly educational workshops, which consisted of short lectures on PA guidelines, PA benefits, and various psychological strategies to promote PA participation and adherence. In educational workshops, participants first learned about PA recommended guidelines, short- and long-term benefits of PA, as well as health consequences of a sedentary lifestyle. Based on the SCT constructs, participants also learned the following psychological strategies for PA participation and adherence: how to effectively set personal goals related to PA (i.e., goal-setting), plan PA activities and monitor PA progress (i.e., planning and self-regulation), increase personal level of motivation for PA adherence (i.e., self-regulation and self-efficacy), mindfully engage in PA (i.e., task self-efficacy), anticipate potential barriers to PA (coping self-efficacy), and utilize appropriate strategies for overcoming potential setbacks in PA adherence (planning and self-regulation). All strategies presented to participants in educational workshops have been identified as important principles of successful behavior adoption. Educational materials were tailored to reflect Filipino cultural values and beliefs related to PA, including qualitative research from previous pilot studies and published literature highlighting psychological and environmental related barriers, and facilitators to PA participation among this population.^13,41^ In addition, participants were also offered a variety of culturally relevant weekly PA classes (i.e., Zumba, line dance, Hip Hop, and strength training) led by certified Filipino instructors for a period of 6 months. The selected PA classes were determined based on previous literature on preferred activities in this population.^13,41^ All educational workshops and PA classes were held in a group setting of the Filipino participants and CHWs. Educational workshops and classes were provided in a mix of English and Tagalog once a week for each session.

### Measures

Prior to starting the Get Phit program, participants completed baseline surveys consisting of seven measures. After completion of the six-month Get Phit program, participants completed the same follow-up surveys. These surveys consisted of the following measures: the Godin Leisure-Time Exercise Questionnaire (GLTEQ),^42^ the Exercise Self-Efficacy Scale (ESES),^43^ the Multidimensional Outcome Expectations for Exercise Scale (MOEES),^44^ the Social Support for Exercise Scale (SSES),^45^ the PA Enjoyment Scale (PACES),^46^ the Exercise Self-Regulation Questionnaire (SRQ-E),^47^ and the Exercise Goal-Setting Scale (EGS).^48^ Every measure has previously shown high construct validity in clinical and experimental settings and strong reliability.^42-48^ In the current study, the overall alpha levels for each subscale and total scale on all measures were greater than the recommended 0.80 value, ranging from 0.81 to 0.96 (Table 1). Due to high reliability, all measures were included in the data analysis. During the course of intervention, weekly journal entries were made to track participants’ attendance of educational workshops and PA classes.In addition, participants completed a 7-item demographic questionnaire to assess their age, gender, level of education, employment status, marital status, number of children, and country of birth. Following intervention, participants were asked to rate their satisfaction with each component of the program.

*Behavior measure*

Physical Activity: The GLTEQ^42^ is a four-item self-report that provides the frequency of strenuous-, moderate-, and light-intensity PA engagement for more than 15 minutes during a 7-day period. Scores higher than 24 on this measure categorize an individual as highly physically active, while scores between 14 and 24 indicate moderately PA levels. Scores below 14 on this measure suggest that an individual is insufficiently active or lives a sedentary lifestyle. Previously, the GLTEQ has shown validity, reliability, and sensitivity to change in physically active and insufficiently active adult groups.^42,49^

*Psychosocial measures*

Exercise Self-efficacy: The ESES^43^ is an 8-item self-report that assesses individuals’ beliefs in their abilities to continue exercising on a three time per week basis at moderate intensities for 40+ minutes per session. The scale items are measured on a 10-point Likert scale with anchors ranging from 0 (not confident) to 10 (very confident). Higher scores on this measure are indicative of higher levels of confidence in an individual’s ability to maintain the behavior.

Outcome Expectations: The MOEES^44^ is a 15-item self-report questionnaire that explores individuals’ beliefs or expectations about the physical and social benefits of regular PA. It includes three subscales: Physical Outcome Expectation Subscale (6 items), Social Outcome Expectation Subscale (4 items), and Self-Evaluative Outcome Expectation Subscale (5 items). The scale items are measured on a 5-point Likert scale, ranging from 1 (strongly disagree) to 5 (strongly agree). This measure does not provide a composite score. However, higher scores on each subscale indicate higher expectations in regards to physical, social, and self-evaluative outcomes related to PA.

Social Support: The SSES^45^ is a 16-item self-report questionnaire that reflects how often friends and family members of the person who is trying to exercise regularly say or do something supportive or discouraging. The scale items are measured on an 8-point Likert scale ranging from 1 (none) to 8 (does not apply). Higher scores on the SSES scale items are indicative of stronger social support from the individual’s family or friends in the proess of a behavior change.

Physical Activity Enjoyment: The PACES^46^ is an 18-item self-report questionnaire that assesses individuals’ feelings at the moment about their ongoing PA. The scale items are measured on a 7-point Likert scale, where higher scores reflect greater levels of enjoyment. Eleven items of the PACES are reverse scored.

Self-regulation: The SRQ-E^47^ is a 16-item self-report that explores individuals’ reasons for why they choose to exercise regularly, including personal reasons and societal pressure. The SRQ-E comprises the following four subscales that measure different types of motivation for PA: External Regulation Subscale, Introjected Regulation Subscale, Identified Regulation Subscale, and Intrinsic Motivation Subscale. The SRQ-E items are measured on a 7-point Likert scale, where subscale scores reflect the relative impact of intrinsic and extrinsic factors in a person’s motivation to be physically active.

Goal setting: EGS^48^ is a 14-item self-report that assess an individual’s ability to independently set exercise goals and plan exercise activities. The EGS is measured on a 5-point Likert scale, ranging from 1 (does not describe me) to 5 (describes completely). Higher scores on this measure are indicative of an individual’s higher ability to set exercise-related goals and develop an exercise regimen.

### Statistical analysis

The IBM SPSS Statistics software (version 24.0; https://www.ibm.com) was used for data analysis. First, descriptive statistics, including the mean, standard deviation (SD) and/or frequency as well as percentage were calculated for each demographic variable. Second, summary and mean subscale scores were calculated for each measure. Alpha coefficients were computed for each subscale and total scale of all survey measures to determine the internal consistency and reliability of the data. The recommended Cronbach’s alpha level used for ensuring high internal consistency of the items was 0.80.^50^ Prior to running *t* tests, a Shapiro-Wilk test was conducted to determine whether the variables were normally distributed. This assumption was violated for the GLTEQ, the ESES,and the SRQ-E. Thus, Wilcoxon signed-rank tests were performed to assess the pre- and post-intervention changes for the following outcome variables: weekly PA participation; exercise self-efficacy; and exercise motivation. Further, dependent-samples *t* tests were conducted to identify the mean differences in pre- and post- intervention scores for the remaining outcome variables: exercise outcome expectations; exercise social support; PA enjoyment; and exercise goal-setting. *P* values < 0.05 were considered significant in all analyses.

## Results

In total, 44 participants were enrolled in the study. By mid-intervention (May of 2018), 7 participants (16%) withdrew from the study due to scheduling conflicts, family or work obligations, or medical illness. Thus, a total of 37 participants (21 females, 56.8%; and 16 males; 43.2% males) completed the intervention and the pre-/post-intervention assessment, resulting in an 84% retention rate. Six monthly educational workshops and 26 weekly PA classes were offered during the 6 months of intervention. PA classes took place on weeknights and weekends from once a week, while educational workshops were held on weekends of each month. Attendance of monthly educational workshops ranged from 94% to 100%. Attendance and completion of weekly PA classes ranged from 92% to 100%. At the conclusion of the intervention, participants reported their satisfaction rates for each component of the program, where 97% of participants were highly satisfied with educational workshops and 95% were highly satisfied with PA classes. Based on the baseline reports for the Godin-Leisure Time Questionnaire, 13 participants (35.1%) were classified as physically active, 10 participants (27%) were moderately active, and 14 participants (37.8%) were insufficiently active or lived a sedentary lifestyle. Descriptive analyses (Table 2) indicated that participants’ ages ranged from 24 to 55 years old (M = 56.1; SD = 10.8). The majority of the participants were married (78.4%) and Philippine-born (86.5%), had an associate’s or bachelor’s degree (62.2%) and 1-2 children (54.1%). The majority of the participants were full-time employees (83.8%). Ten participants (27%) worked in a health-related occupation.

As seen in Table 3, Wilcoxon signed-rank *t* tests indicated significantly higher follow-up median scores on the following measures and subscales: Godin Leisure-Time Exercise Scale (*Z* = -3.24, *P* < 0.001), ESES (*Z* = -4.37, *P* < 0.001), Identified Regulation (*Z* = -.3.46, *P* < 0.001), and Intrinsic Motivation (*Z* = -3.20, *P* < 0.001). Based on the dependent-samples *t* tests, positive changes were observed for Physical Outcome Expectations (mean difference = 0.23, SD = 0.09, *P* < 0.01), Social Outcome Expectations (mean difference = 0.29, *SD* = 0.13, *P* < 0.01), Self-Evaluative Outcome Expectations (mean difference = 0.43, SD = 0.32, *P* < 0.01), Physical Activity Enjoyment (mean difference = 0.74, SD = 0.36, *P* < 0.01), Social Support for Exercise Scale – Family (mean difference = 0.74, SD = 0.35, *P* < 0.01), Social Support for Exercise – Friends (mean difference = 0.73, SD = 0.33, *P* < 0.01), and Exercise Goal-Setting (mean difference = 0.50, SD = 0.28, *P* < 0.01). Results for the dependent-samples t-tests are shown in Table 4. In addition, 11 out 14 participants (78.6%), who were classified as insufficiently active at baseline, reported moderate PA levels following intervention. Similarly, 6 out of 10 participants (60%), who were moderately active at baseline transitioned into the highly active group based on the post-intervention reports.

## Disscussion

The study findings illustrated increased levels of weekly PA participation among Filipino Americans in the Greater Philadelphia Area following the Get Phit intervention. Specifically, 78.6% of participants in the study, who lived a sedentary lifestyle prior to intervention, increased their weekly PA to moderate levels following intervention. Additionally, 60% of participants in the sample, who were moderately active at baseline, reported highly active PA levels after six months of Get Phit. Furthermore, the findings showed significant increases in exercise-related psychosocial factors, indicating positive changes in participants’ attitudes and views of PA. Specifically, participants reported increased levels of exercise motivation, exercise enjoyment, and exercise self-efficacy, the latter of which signified higher levels of confidence in participants’ abilities to independently engage in PA.

In regard to motivation, participants exhibited positive changes in intrinsic motivation and identified regulation, which are the two intrinsic forms of one’s self-determination to carry out a behavior. This finding reflects participants’ increased interest in PA participation and PA enjoyment following intervention. This result also indicates positive changes in participants’ views of PA, awarding a conscious value to this behavior. On the contrary, no changes were found in participants’ external and introjected regulations, which are the two forms of extrinsic motivation. This can be explained by participants’ initial motivation for participation in the intervention. Specifically, participants reported higher scores on the two intrinsic forms of motivation mentioned previously, as compared to scores on the two forms of extrinsic motivation. It is plausible that our participants were not initially driven to participate in the study by external sources, such as rewards, feelings of pride, avoidance of punishment, or avoidance of negative emotions (e.g., frustration, guilt, shame).

Furthermore, participants gained an increased understanding of the physical and social benefits of PA following the intervention. Participants also reported an increased ability to independently set exercise-related goals and utilize appropriate strategies for goal attainment. Lastly, participants indicated having stronger social support from family and friends who not only encouraged participants to continue their PA involvement, but also expressed the desire to exercise jointly. Some of the participants’ family members and friends voluntarily participated in a number of educational workshops and PA classes for moral support.

### Implications for practice and research

The current study expands on the literature that has demonstrated positive changes in PA levels and perceived benefits following an intervention program among Asian Americans.^31,51^ Existing literature on culturally tailored interventions have seldom implemented both educational and behavioral based components. For instance, an 18-month nutrition and PA intervention implemented among 337 Filipino Americans in San Diego county, California, resulted in increased levels of PA and adoption of a low-weight diet.^25^ Nevertheless, the majority of such studies have been conducted among Chinese Americans.^31,51^ These studies used heterogeneous methods in their intervention development, tailoring, and implementation, which produced inconsistent results and created gaps in the literature. The positive findings of the current study attest to the feasibility of engaging adult Filipino Americans in a culturally tailored PA intervention. In addition, high attendance rates and favorable ratings of satisfaction with various components of the program, indicate increased PA enjoyment and motivation among participants. However, recruitment, retention, and post-intervention assessment data highlight the need to account for a 14% participant loss when determining appropriate sample size in future studies.

To our knowledge, this was the first study that tested the feasibility of a culturally tailored PA intervention designed specifically for the needs and preferences of Filipino Americans, an ethnic Asian minority population. In addition, this study evaluated the changes in PA levels and psychosocial outcomes related to PA following intervention. Psychosocial determinants are known to reflect exercise adherence levels and should be incorporated in PA interventions.^51,52^ Furthermore, the Get Phit intervention served as a culturally relevant program for the Filipino community, which promoted regular exercise participation. Unique features of this intervention, such as having instructors of the same ethnicity, providing educational workshops on PA by CHWs, as well as offering a variety of culturally relevant PA classes, were the major strengths of this study. These features helped us create a culturally-sensitive and inclusive environment, where participants gained a sense of relatedness and comfort. CHWs can aid in improving adherence and positive outcomes of programs in underserved minority populations.

The present study included several limitations. First, due to the participant drop out in the course of the intervention, the final sample size was small. The small sample size precluded mediation analyses between the psychosocial and PA variables. Therefore, future studies would benefit from increasing sample size. Interventions with a significantly larger sample size could have shown a clearer pattern of changes in PA adherence and PA related outcomes. Second, the pre-test and post-test research design of this study resulted in difficulty establishing cause and effect relationships between the Get Phit intervention, PA outcomes, and psychosocial variables. Thus, future experimental studies with a control group are needed to further investigate changes in PA levels and psychosocial outcomes following the same intervention. Additionally, self-report PA measures were utilized in the study. Although the GLTEQ has shown validity,reliability, and sensitivity to change among diverse groups, participants may have exaggerated their reported levels of weekly PA, as well as answered other survey questions in a biased manner. Hence, future studies should also incorporate objective measures of PA (e.g., pedometers or accelerometers) to avoid biases associated with self-report. With studies that implemented culturally tailored interventions, few have attempted to identify which cultural characteristics led to the effectiveness of the intervention. Since our study had incorporated several culturally tailored components, it was difficult to identify specifically which aspects were successful. Thus, future qualitative follow-up with participants to obtain feedback is needed.

## Conclusion

In summary, designing and implementing PA interventions in Filipino Americans could serve as a pivotal step for improving their physical health and psychosocial outcomes related to PA. Filipino Americans remain a highly understudied population who can combat high prevalence of chronic diseases within the community with the help of PA interventions. Despite the limitations highlighted previously, the positive findings from the current study indicate that this community-based PA intervention has the potential to increase PA engagement and related psychosocial outcomes. The significant findings and lessons learned from this community-based study set the stage for a larger randomized control trial that will control for threats to internal validity. Future studies can also utilize objective measures to fully assess the efficacy of Get Phit in increasing PA levels and improving health-related outcomes among Filipino and other Asian Americans in various geographic regions.

## Acknowledgments

The authors would like to thank the community health workers, leaders, and community members from the Philippine Community of Southern New Jersey who were essential to implementing the program.

## Funding

The project was supported by the Association for Applied Sport Psychology Community Outreach Grant. This project was partially supported by TUFCCC/HC Regional Comprehensive Cancer Health Disparity Partnership, Award Number U54 CA221704(5) from the National Cancer Institute of National Institutes of Health (NCI/NIH). Its contents are solely the responsibility of the authors and do not necessarily represent the official views of the NCI/NIH.

## Competing interests

None.

## Ethical approval

Temple University’s Institutional Review Board (IRB) granted approval for this study. Written informed consents were obtained from participants prior to participating in the study.

## Authors’ contributions

ABh developed the study, oversaw the intervention, and manuscript preparation; KP contributed to preparing the manuscript and data analysis; ABe, JD, and NSJ assisted with intervention development and carrying out the intervention; MS and GXM contributed to the study development and management.

**Table 1:** Internal consistency estimates of subscales

**Scale**	**Subscale**	**Number of items**	**Cronbach’s α**
Godin Leisure-Time Exercise Questionnaire (GLTEQ)		4	0.85
Exercise Self-Efficacy Scale (ESES)		8	0.90
Multidimensional Outcome Expectations for Exercise Scale (MOEES)	Physical Outcome	6	0.92
	Social Outcome	4	0.91
	Self-Evaluative Outcome	5	0.92
Social Support for Exercise Scale (SSES)	Social Support – Family	8	0.81
	Social Support - Friends	8	0.82
PA Enjoyment Scale (PACES)		18	0.93
Exercise Self-Regulation Questionnaire (SRQ-E)	External Regulation	4	0.95
	Itrojected Regulation	4	0.93
	Identified Regulation	4	0.95
	Intrinsic Regulation	4	0.94
Exercise Goal-Setting Scale (EGS)		14	0.96

**Table 2:** Demographic characteristics among study sample (n = 37)

**Variable**		
Age (years), mean (SD)		56.1 (10.8)
Gender, No, (%)	Men	16 (43.2)
	Women	21 (56.8)
Marital status, No, (%)	Single	5 (13.5)
	Married	29 (78.4)
	Divorced	3 (8.1)
Educational level, No, (%)	High school graduate	3 (8.1)
	Associate’s or bachelor’s degree	23 (62.2)
	Master’s of doctorate degree	11 (29.7)
Children, No, (%)	Yes	32 (86.5)
	No	5 (13.5)
Number of children, No, (%)	0	5 (13.5)
	1-2	20 (54.1)
	3+	12 (32.4)
Employed outside of home, No, (%)	Yes	28 (75.7)
	No	4 (10.8)
Hours worked per week, No, (%)	Do not work	5 (13.5)
	Less than 40	1 (2.7)
	40 or above	31 (83.8)
Health related occupation, No, (%)	Yes	10 (27)
	No	27 (73)
Born in the Philippines, No, (%)	Yes	32 (86.5)
	No	5 (13.5)
Physically active^a^, No, (%)	40.06 (4.53)	13 (35.1)
Moderately active^a^, No, (%)	16.62 (1.34)	10 (27)
Insufficiently active^a^, No, (%)	7.78 (2.43)	14 (37.8)

^a^ Based on the baseline scores for the Godin Leisure-Time Exercise Questionnaire.

**Table 3:** The Wilcoxon signed-rank pre- and post-test results for physical activity and psychosocial outcome measures (n = 37)

**Scale/ Subscale**	**Median percent change**	***P*** ** value**	**Effect size**
Physical Activity Levels^a^	68.75%	<0.001	0.45
*Psychosocial measures*			
Exercise Self-Efficacy^b^	7.30%	<0.001	0.51
Exercise Motivation^c^			
External Regulation	-0.33%	0.33	0.11
Introjected Regulation	3.00%	0.41	0.10
Identified Regulation	5.50%	<0.001	0.40
Intrinsic Motivation	5.25%	<0.001	0.37

^a^ Scores > 24 indicate high PA levels; scores between 14 and 24 indicate moderate PA levels; scores < 14 indicate insufficiently active lifestyle. ^b^Total possible score for this measure is 10. ^c^ Total possible score for each subscale is 7.

**Table 4:** Dependent-samples pre- and post-test results for psychosocial outcome measures (n = 37)

**Scale/ Subscale**	**Mean Difference** **(M; SD)**	**95% CI** ^a^
PA Outcome Expectations^b^		
Physical Outcome	0.23 (0.09)	(0.20, 0.26)
Social Outcome	0.29 (0.13)	(0.25, 0.33)
Self-Evaluative Outcome	0.43 (0.32)	(0.32, 0.54)
Physical Activity Enjoyment^b^	0.74 (0.36)	(0.62, 0.86)
Social Support – Family^c^	0.74 (0.35)	(0.62, 0.86)
Social Support – Friends^c^	0.73 (0.33)	(0.62, 0.84)
Exercise Goal-Setting^d^	0.50 (0.28)	(0.41, 0.59)

^a^Confidence intervals for every subscale were significant at the.01 level. ^b^Total possible scores for these subscales is 7. ^c^Total possible score for this measure is 8. ^d^Total possible score for this measure is 5.
